# Acoustic discrimination in the grey bamboo shark *Chiloscyllium griseum*

**DOI:** 10.1038/s41598-022-10257-1

**Published:** 2022-04-20

**Authors:** Tamar Poppelier, Jana Bonsberger, Boris Woody Berkhout, Reneé Pollmanns, Vera Schluessel

**Affiliations:** 1 Department of Comparative Sensory Biology and Neurobiology, Institute of Zoology, University of Bonn, Meckenheimer Allee 169, 53115 Bonn, Germany; 2 FNWI, University of Amsterdam, Science Park 904, 1098 XH Amsterdam, The Netherlands

**Keywords:** Zoology, Animal behaviour, Perception

## Abstract

Cognitive abilities of sharks are well developed and comparable to teleosts and other vertebrates. Most studies exploring elasmobranch cognitive abilities have used visual stimuli, assessing a wide range of discrimination tasks, memory retention and spatial learning abilities. Some studies using acoustic stimuli in a cognitive context have been conducted, but a basic understanding of sound induced behavioural changes and the underlying mechanisms involved are still lacking. This study explored the acoustic discrimination abilities of seven juvenile grey bamboo sharks (*Chiloscyllium griseum*) using a Go/No-Go method, which so far had never been tested in sharks before. After this, the smallest frequency difference leading to a change in behaviour in the sharks was studied using a series of transfer tests. Our results show that grey bamboo sharks can learn a Go/No-Go task using both visual and acoustic stimuli. Transfer tests elucidated that, when both stimulus types were presented, both were used. Within the tested range of 90–210 Hz, a frequency difference of 20–30 Hz is sufficient to discriminate the two sounds, which is comparable to results previously collected for sharks and teleosts. Currently, there is still a substantial lack of knowledge concerning the acoustic abilities and sound induced behaviours of sharks while anthropogenic noise is constantly on the rise. New insights into shark sound recognition, detection and use are therefore of the utmost importance and will aid in management and conservation efforts of sharks.

## Introduction

Sharks have a wide range of cognitive abilities, which are comparable to teleosts and other vertebrates^1^. Examples of cognitive abilities are recognition, discrimination and social learning functions. These abilities likely aid sharks in predator avoidance, social interactions, foraging and/or habitat selection^2^. Learning is most simply defined as a change in behaviour as a result of experience^3^. It is an important mechanism of adaptation to environmental unpredictability^3^ and can increase an individual’s chances of maximizing its fitness^4^. One of the first studies looking at the ability of sharks to learn involved an operant conditioning regime^5^. In this study, sharks were successfully trained to acquire food by pressing a submerged bell, clearly showing that sharks are able to perform a simple cognitive task. Since then, a range of different studies have followed, mostly focusing on visual discrimination abilities (for reviews see^1,2,6^). For example, grey bamboo sharks *Chiloscyllium griseum* can visually discriminate a wide range of stationary and moving two-dimensional objects, recognize symmetry, perceive subjective and illusory contours and even form mental categories^7–13^. Furthermore, memory retention and spatial learning abilities were found in both grey bamboo sharks, freshwater stingrays *Potamotrygon motoro*, Port Jackson sharks *Heterodontus portusjacksoni* and coral cat sharks *Atelomycterus marmoratus*^12,14–18^. Small spotted catsharks *Scyliorhinus canicula* can perform a foraging task using electroreceptive stimuli^19^, indicating that not only visual stimuli can be used researching cognitive abilities. Several studies showed that sharks are able to be conditioned to an acoustic stimulus. However, cognitive abilities of sharks using acoustic stimuli have barely been researched in depth^20,21^, and consequently, a basic understanding of sound induced behavioural changes and the underlying mechanisms involved in this process is still lacking.

Underwater sound can be a highly informative cue. It travels further than light and faster than olfactory cues. It is also highly directional, further increasing the information contained in the cue^22,23^. It can be categorized into two different components: sound pressure and particle motion. Sound pressure is described as a deviation of the ambient pressure caused by a sound wave, and particle motion results from the oscillatory displacement of particles within a propagating sound wave^24^. The source of underwater sound in marine environments (from here on ‘sound’) can either be natural or anthropogenic. Abiotic natural sound is mostly generated by wind, waves, and tectonic processes. Biotic natural sound is generated by a variety of marine organisms, such as snapping shrimp, sperm whales communicating through echolocation or a male red grouper courting a female^25–27^. Anthropogenic sound is any sound produced by human activity, such as container shipping, drilling or military sonar, and is often classified as either acute (high intensity, short duration, and often pulsed) or chronic (long term and low intensity)^28^. Anthropogenic sound often differs from natural sound in acoustic characteristics such as sound pressure level, impulsiveness and repetition rate^28^. Regardless of its nature, sound influences the life and behaviour of marine organisms and its effects likely vary with acoustic characteristics of sound (source, duration, level, spectrum, etc.) and acoustic sensitivity of the receiving animals.

In bony fishes, sound plays and important role^29–31^. For many species, sound is implicated in social interactions such as reproductive or territorial behaviour and has even been suggested to aid in social aggregation (e.g. shoaling)^32–34^. Moreover, natural sound is used for navigation^29,31^ and locating predators or prey^35,36^. To be able to determine the type (e.g., predator or prey) or location of a sound source, a fish must successfully discriminate and identify different sounds. This ability has been shown for example in goldfish *Carassius auratus*^37,38^, larval reef fish^29,39^ and sound producing electric fish *Pollimyrus adspersus*^40^. Bony fishes can typically detect low frequencies up to 3 kHz, but many species are most responsive to sounds below 1 kHz^41^. Sharks are most sensitive to low frequency sounds between 20 Hz and 1 kHz^42^. They do not seem to produce sound, but like most bony fishes, sharks can detect the (directional) particle motion component of sound^36,42^. A small opening on each side of the head directly leads into the inner ear, where particle motion induces bending of the cilia of sensory hair cells in the ear. This in turn generates a physiological response resulting in the detection of sound^42,43^. A second system contributing to the detection of sound is the lateral line system. The lateral line in elasmobranchs can detect low frequency sounds between 1 and 200 Hz and has the greatest sensitivity between 20 and 30 Hz^44,45^. This system is also stimulated by back-and-forth movement of cilia, and is considered a short-distance sensory system (one to two body lengths)^43,46,47^. Sharks are generally considered not to be able to detect the sound pressure component due to their lack of a pressure-to-displacement transducer like a swim bladder or other air-filled cavity^36^. However, two studies showed that species without an apparent pressure-to-displacement transducer (triplefin larvae (family: Tripterygiidae) and grey bamboo sharks) can detect both particle motion and sound pressure^29,48^. This suggests that an additional sensory mechanism might be involved in detecting sound pressure in some species. A potential candidate for the detection of sound pressure in sharks is the lateral line system, but such a function has yet to be experimentally demonstrated.

Although a proper understanding of the use of sound in sharks is lacking, published accounts of sharks detecting and being attracted to sound are available^20,36,48–53^. Additionally, previous research on acoustic cognitive abilities showed that lemon sharks *Negaprion brevirostris* successfully discriminated between frequencies (160 vs. 40 Hz, 80 vs. 40 Hz and 70 vs. 40 Hz) in an approach-avoiding experiment, with the smallest detectable difference in this experiment being 20 Hz (60 vs. 40 Hz)^20^. In addition, a recent paper showed that Port Jackson sharks *H. portusjacksoni* were able to learn an association task using an acoustic stimulus^21^. However, a substantial knowledge gap on the acoustic abilities of sharks and their ability to discriminate frequencies remains.

Go/No-Go testing (e.g. an animal touching a lever when a circle is presented, but not touching the lever when a square is presented) is often used as a component of behavioural examination in order to assess inhibitory control^54^. Previously, Go/No-Go tasks have been successfully used as a measure of learning in bony fishes^55,56^ and of judgement bias, impulsiveness and behavioural inhibition in mammals^57–61^.

In the present study it was tested whether grey bamboo sharks can perform a Go/No-Go task based on sound and whether they can discriminate between two frequencies. This was accomplished by training sharks to associate one tone with action A and another tone with action B. After successful training it was tested whether sharks associated frequencies between 90 and 210 Hz with behaviour A or B.

## Method

### Subjects

Sixteen naïve juvenile grey bamboo sharks (*C. griseum,* eight females and eight males ranging between 63–75 cm (Group 1; experiments in 2018–2019) and 39–45 cm [Group 2; experiments in 2020–2021) total length (TL)] were obtained from Haus des Meeres (Vienna) where they were bred in captivity. The sharks were housed in separate aquarium systems (L × W × H: tank 1 and 2: 1.6 × 0.5 × 0.5 m; tank 3: 1.8 × 0.5 × 0.5 m; tank 4: 1.8 × 0.9 × 0.6 m; tank 5 and 6: 1 × 1.75 × 0.5 m) with either two or three sharks per tank. All aquaria were filled with filtered saltwater [conductance: about 50mS (ca. 10,217 kg/dm^3^)] at ± 25 °C. The systems were aerated with air stones and the animals were kept on a 12 h day:12 h night cycle. Regular water testing was done to ensure constant environmental conditions (salinity, temperature, NO_2_, KH and pH). Environmental conditions were close to identical throughout the different aquarium systems. The experimental tank was connected to the main aquarium system to ensure stable environmental conditions. Individuals were identified by phenotypic characteristics (e.g., colour, sex and size). Sharks were fed exclusively during experimental sessions. Food consisted of shrimps, mussels, fish and squid. Once a week, vitamin supplements (JBL Atvitol) were added to the feed.

### Experimental set-up

**Figure 1:**
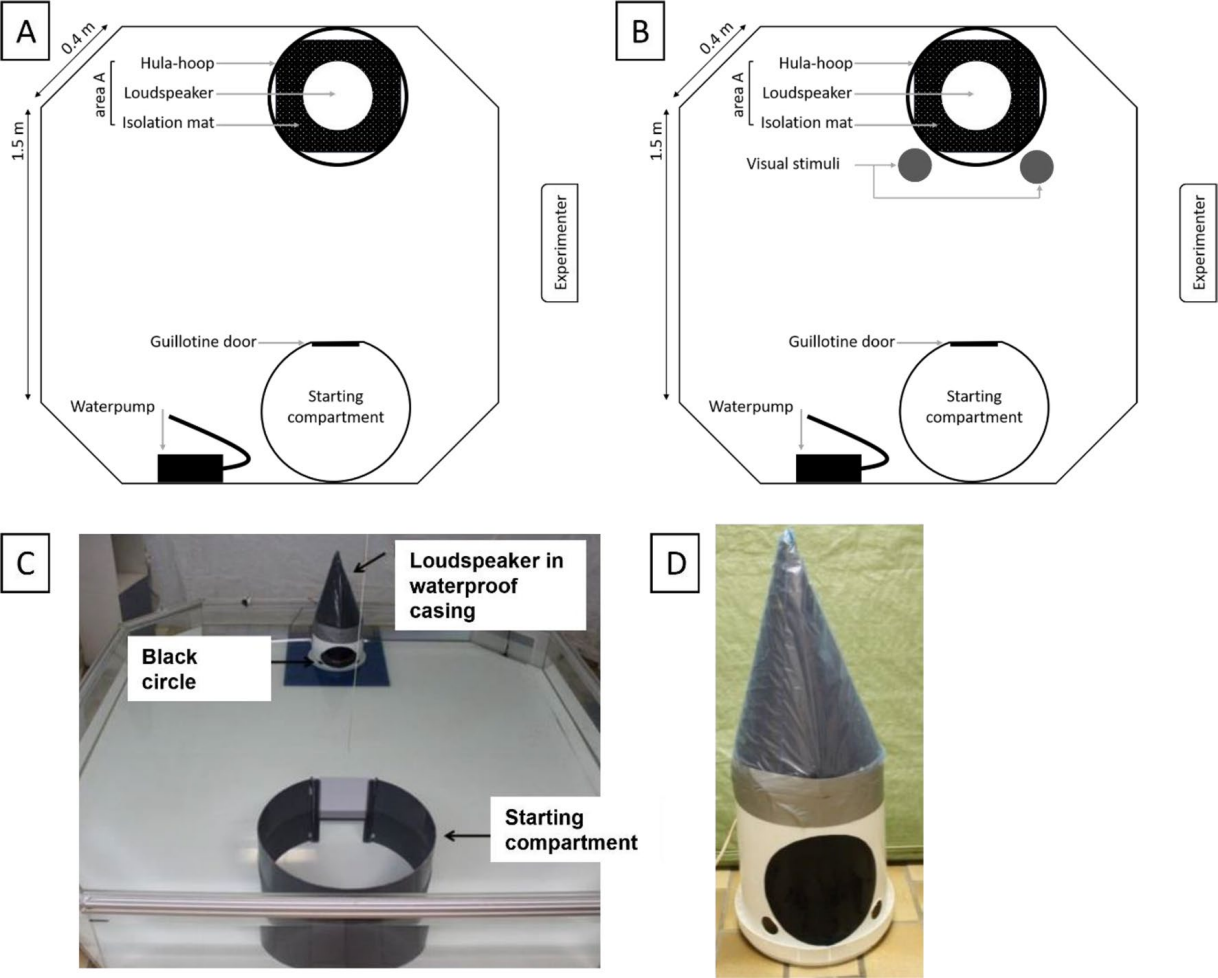
Schematic overview of experimental set-up for ‘Go/No-Go’ experiments (not to scale). (**A**) ’No-Go’-trial (visual stimulus absent). (**B**) ‘Go’-trial (visual stimuli present for Group 2). (**C**) Side view of experimental setting. Note that the water pump was taken out for this picture. (**D**) Loudspeaker built into waterproof casing with a black circle on the outside.

Experiments were carried out in an octagonal tank of 2.1 × 2.1 × 0.35 m (Fig. 1) which was connected to the main aquarium system. A white tarp was placed around the area of the experimental tank to minimise visual distractions during the experiments. In the beginning of each experimental session, the shark was placed in a black, circular starting compartment (SC) until training commenced (Fig. 1). The SC was opened and closed by a light grey guillotine door which was operated by a manual pulley system. The opening was located on the floor since bamboo sharks are a benthic species. The loudspeaker was placed opposite of the SC guillotine door. An underwater loudspeaker was not available. Therefore, a loudspeaker (Visaton TIW 200XS) was raised above the water surface by a handmade waterproof white casing and connected to a Harman Kardon HK 6200 amplifier. The bottom of the waterproof casing rested on a blue filtration mat to minimise acoustic vibrations being transferred to the tank floor. A black circle (ø 18 cm) was stuck on the front of the waterproof casing which was submerged (Fig. 1). The experimental sounds (Group 1: 140 and 200 Hz; Group 2: 90 and 210 Hz) were selected based on previous research and experiments in this lab. To minimise the chances of giving unintentional cues, a webcam (Logitech HD Pro Webcam C920) was installed above the experimental tank to observe the shark during the experiment without looking at it directly. All sessions were recorded with this webcam.

### Acoustic measurements

**Figure 2:**
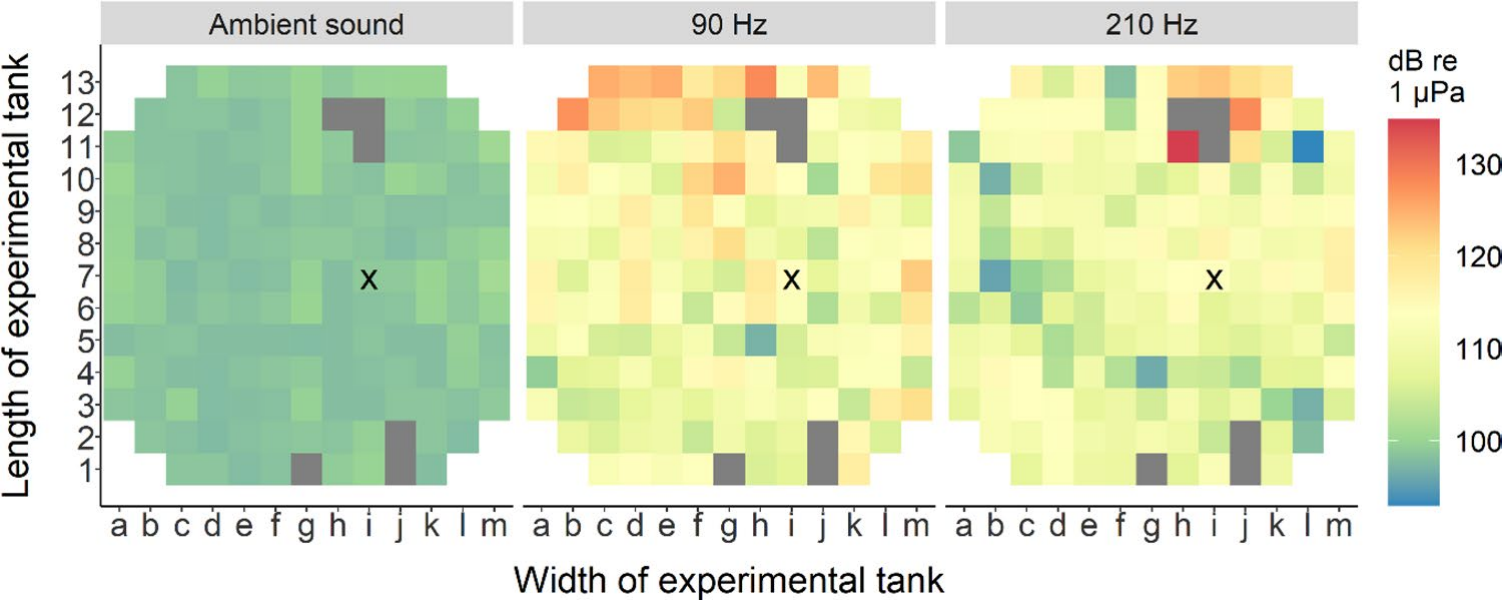
Sound pressure levels (SPL; dB re 1 µPa) in the experimental tank. Measurements were made along a 15 cm grid, with distance from tank walls being 13 cm. Grey tiles indicate that no measurement was made at that grid point due to the loudspeaker (h12, i11 and i12) and SC (g1, j1 and j2) blocking that position. Grid point ‘i7’ is marked with an ‘x’.

Ambient sound (day to day sounds originating from water in- and outflow, aeration pumps, protein skimmers, etc.) and sound pressure levels (SPL) of the used experimental frequencies (90 and 210 Hz) in the experimental tank were measured using an omnidirectional hydrophone (Aquarian Audio H2a with inbuilt preamplifier, manufacturer-calibrated sensitivity − 180 dB re 1 V/mPa; frequency range 0.01–100 kHz) and the software program Audacity (v.2.4.2;^62^). Calibration of the hydrophone was achieved following the freely available online calibration protocol from Aquarian Hydrophones. A function generator (Voltcraft FG-506) and an oscilloscope (Yokogawa DL1300A) were used as signal generator and output measuring device, respectively. Sound pressure level of each sound (ambient sound, 90 Hz and 210 Hz) was measured three times for 5 s on 151 unique locations in the experimental tank (along a 15 cm grid). Heatmaps of each sound were made using the average of each sound per location (Fig. 2). For the heatmap of ambient sound, SPL represents the loudest frequency present (40 Hz). Spectrograms of ambient sound, 90 Hz and 210 Hz tones can be found in the Supplementary Information (SI 2). All other tones used during transfer tests (100–200 Hz) were measured at one location in the experimental tank.

### Experimental procedure

The commonly used method for testing discriminatory abilities in sharks is a two-alternative forced choice test. However, to test acoustic discrimination abilities, the experimental design of a two-alternative forced choice task would have required two loudspeakers playing a sound at the same time. Due to the small size of the experimental tank, emitted sound waves would have reflected and interfered with correct sound localization. Secondly, sharks often exhibit side preferences (pers. comm. Vera Schluessel). Therefore, a Go/No-Go task, a method never before tested in sharks, was preferred over a two-alternative forced choice experiment.

#### Acclimatization and pre-training

Before (pre-)training commenced, sharks were familiarized with the experimental tank. In the first step, individual sharks were transferred to the experimental tank for roughly 10 min and allowed to rest or swim freely during this time. It also permitted sharks getting used to being handled and getting fed with black, plastic forceps. This period ended after four days when sharks moved and fed without hesitation. In a second step, the starting compartment, guillotine door and loudspeaker were added to the experimental tank to get the sharks used to the experimental setup. Sharks that did not swim or eat in the experimental tank did not progress to pre-training.

#### Training

The two groups of individuals (total of N = 16) were each trained in a different procedure and by a different trainer in two separate years. Group 1 consisted of seven animals out of which four individuals successfully learned. Group 2 consisted of nine animals out of which three individuals successfully learned.

### Group 1

After acclimatizing the sharks, individuals were pre-trained to touch the loudspeaker in case of a 200 Hz tone (‘Go’ trial). There were 10 consecutive trials per day, which formed a session. Sessions were run daily for six days per week. An animal was considered to have learned the task in this step when reaching learning criterion (LC). LC for this step was established at a minimum of 90% correct choice for three consecutive sessions.

In a second step, individuals were then taught not to touch the loudspeaker when no tone was played (‘No-Go’), but still touch it when a 200 Hz tone was played. In this step, so called super sessions (SS) were performed, which consisted of 45–120 trials. The trial number was open towards the end, i.e. the animal continued to participate in the session until it was completed. Individuals were required to make consecutive correct choice(s) during sets of played frequencies. Super sessions were performed at a maximum of three days apart, and no experimental sessions were performed on the days in between. Once completed successfully, sharks progressed to a third experimental step.

**Figure 3:**
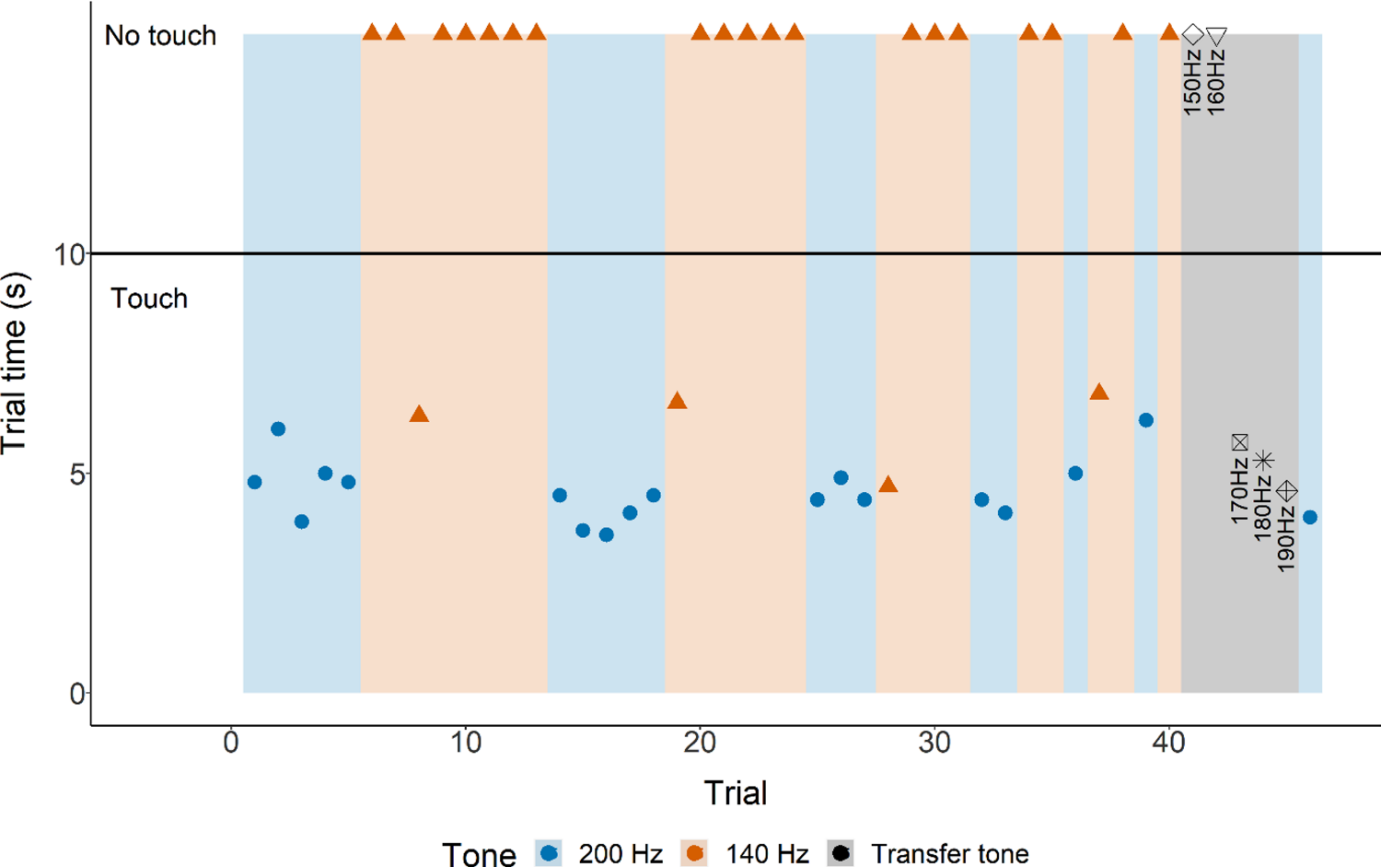
Individual supersession data for shark B. Touching the loudspeaker indicated by the time it took to touch (LST time). Not touching the loudspeaker is indicated by the shapes (at 15 s, indicating a capped trial i.e., the loudspeaker was not touched) above the horizontal solid black line (indicating the maximum trial time for a ‘Go’-trial). Background colour indicates each separate phase (‘Go’ phase: blue, ‘No-Go’ phase: ‘orange, transfer: grey).

**Figure 4:**
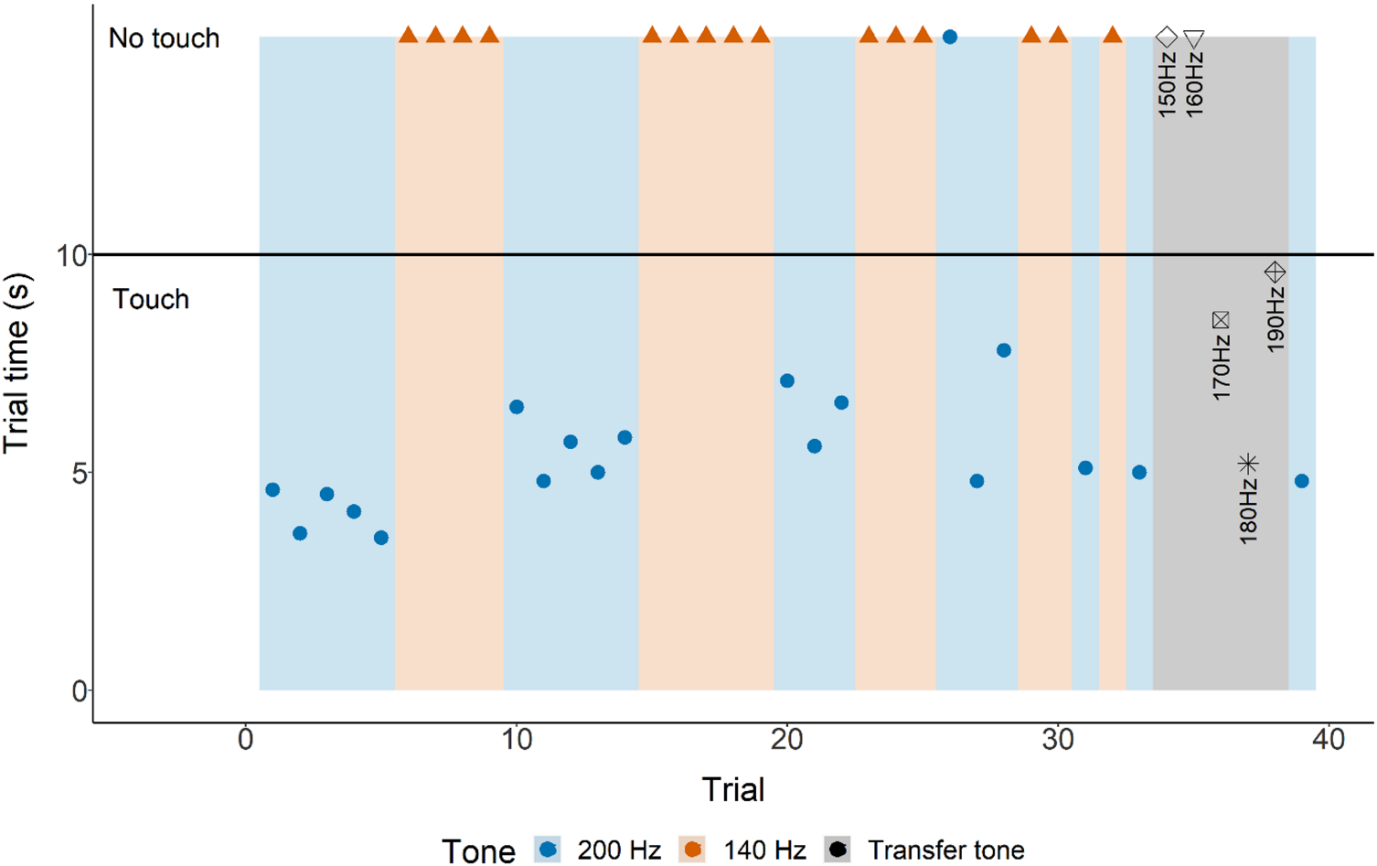
Individual supersession data for shark D. Touching the loudspeaker indicated by the time it took to touch (LST time). Not touching the loudspeaker is indicated by the shapes (at 15 s, indicating a capped trial i.e., the loudspeaker was not touched) above the horizontal solid black line (indicating the maximum trial time for a ‘Go’-trial). Background colour indicates each separate phase (‘Go’ phase: blue, ‘No-Go’ phase: ‘orange, transfer: grey).

In this third step, the Go/No-Go discrimination task was tested using the same 200 Hz tone to indicate a ‘Go’-trial and a 140 Hz tone (instead of no tone) for a ‘No-Go’-trial. Again, so called super sessions (SS) were performed, which consisted of 40–80 trials with trial number open towards the end. Individuals were required to make consecutive correct choice(s) during sets of played frequencies (see Figs. 3 and 4 for the structure of these tests). Again, super sessions were performed at a maximum of three days apart, and no experimental sessions were performed on the days in between.

In all three experimental steps, each trial started by playing a tone, opening the guillotine door, and starting the timer at the same time. For a ‘Go’-trial (200 Hz), a choice was considered to be correct when an individual touched the black circle on the loudspeaker with its snout within 10 s of leaving the SC (action A). When a correct choice was made, food was provided at the black circle. A choice was considered to be incorrect when an individual did not touch the black circle within 10 s of leaving the SC. For a ‘No-Go’-trial (no tone or 140 Hz), a choice was considered to be correct when an individual swam out but did not touch the loudspeaker within 15 s (action B). In step 2, ‘No-Go’-trials were unrewarded regardless of the action performed. In step 3, ‘No-Go’-trials were rewarded. In case of a correct choice, food was provided at the respective location of the shark at that time. A choice was considered to be incorrect when an individual touched the black circle on the loudspeaker.

Amplitude, i.e. sound pressure level, was varied randomly throughout trials to prevent sharks from using this as the deciding cue, instead of frequency.

#### Transfer tests

Following the successful learning of the Go/No-Go task, unfamiliar frequencies were introduced during a super session to determine the smallest difference between the used experimental frequencies that grey bamboo sharks could still discriminate between (from here on: ‘difference threshold’). For this purpose, a super session was carried out in the same manner as described above, but presenting unfamiliar frequencies (150–200 Hz, increments of 10 Hz) during the last six trials.

### Group 2

For the second group, a different approach was used to teach the discrimination task. Two types of stimuli were used to increase the likelihood of the sharks learning the Go/No-Go discrimination task: a visual and an acoustic stimulus (Part A). The visual stimulus was added early on in the experiments as sharks seemed to have difficulty understanding the task when only acoustic stimuli were presented. After acclimatization, the sharks were pre-trained for the Go/No-Go task by luring them to the correct goal location when the respective stimulus/stimuli was/were presented. After this, sessions were run once daily for six days a week.

For this procedure, sessions consisted of 20 trials with equal numbers of ‘Go’ and ‘No-Go’ trials. Each trial started by playing the tone, opening the guillotine door, and starting the timer at the same time. In case of a ‘Go’-trial, a 210 Hz tone was played and a visual stimulus (two upside-down terracotta pots (ø 17 cm) placed on either side of the loudspeaker) was present. A choice was considered to be correct when an individual touched the black circle on the loudspeaker with its snout within 10 s of leaving the SC (action A). When a correct choice was made, food was provided at the black circle. A choice was considered to be incorrect when an individual did not touch the black circle within 10 s of leaving the SC. During a ‘No-Go’-trial, a 90 Hz tone was played, there was no visual stimulus. A choice was considered to be correct when an individual swam out but did not touch the loudspeaker within 10 s (action B). In case of a correct choice, food was provided at the respective location of the shark at that time. A choice was considered to be incorrect when an individual touched the black circle on the loudspeaker. At the start of the experiment the maximum trial time was longer (up to 50 s). As the success rate of the sharks improved, the maximum trial time was gradually reduced (by steps of 10 or 20 s) towards the maximum of 10 s. An animal was considered to have learned the task when reaching learning criterion (LC). LC was established at a minimum of 70% correct choice for five consecutive sessions. Amplitude, i.e. sound pressure level, was varied randomly throughout trials to prevent sharks from using this as the deciding cue, instead of frequency.

After sharks learnt and completed the first set of transfer tests (Transfer 1; see below), the visual stimulus was taken out and normal training sessions resumed to determine again, whether sharks could do the Go/No-Go task with only an acoustic stimulus present (Part B). Rules for correct and incorrect choices, learning criterion and set-up (except for the visual stimulus) remained the same.

#### Transfer tests

##### Transfer 1. Utilized stimuli

The first set of transfer test trials was meant to elucidate which stimulus (acoustic and/or visual) the sharks used to identify the type of trial (‘Go’ or ‘No-Go’). As choice during a transfer test trial was neither correct nor incorrect, transfer test trials were unrewarded. After sharks reached the learning criterion with both acoustic and visual stimuli present, a 90% rewarding scheme was introduced prior to running transfer tests. For the entire transfer test period, food was only provided in a maximum of 18 (out of 20) correct trials, regardless of choice. Which two trials would be unrewarded was randomly determined prior to each session. The 90% rewarding scheme served to prepare the sharks for the subsequent introduction of the unrewarded transfer test trials. This way sharks learnt that a correct choice was not always associated with food and meant to keep sharks from assuming that the unrewarded transfer test trials were ‘incorrect’ and therefore not worth participating in. Transfer trials (Table 1; n = 20 per type and individual) started if performance during the 90% rewarding scheme remained at LC (minimum of 70% correct choice) for three days. Two transfer test trials were then randomly interspersed with the 20 regular trials in a session. The rewarding scheme was changed to 80% (i.e., only a maximum of 16 out of 20 regular trials was rewarded) and number of transfer test trials per session increased to four when performance in regular trials remained unchanged for seven days.

**Table 1:** Transfer 1 trials—utilized stimuli.

Transfer test trial type	Visual stimulus	Acoustic stimulus
A	No	210 Hz
B	Yes	No sound
C	Yes	90 Hz

##### Transfer 2. Difference threshold

A second set of transfer tests aimed to determine what the difference threshold for this group was. A preference for either touching or not touching the loudspeaker (depending on the individual) was detected during training after the visual stimulus was taken out, which could have introduced a bias to the transfer test results, had the same method been used as in Transfer 1. Therefore, using this transfer test method was deemed inefficient and a super session method (similar to the method used for group 2) was used instead.

Again, sharks that reached learning criterion with only the acoustic stimulus present were introduced to an 80% reward scheme (see description above for Transfer 1). If performance remained unchanged, a super session (SS) was initiated. To increase motivation, no session was conducted the day before the SS. Super sessions consisted of 60–80 trials, and individuals were required to make consecutive correct choice(s) during sets of played frequencies (see Fig. 9 for the structure of these tests). In case of an incorrect choice, the set was restarted until the animal reached the required amount of consecutive correct choices. In the final phase, unfamiliar frequencies (100–200 Hz, in increments of 10 Hz) were presented in a random order. Normal trials were unrewarded 80% of the time. Transfer trials with unfamiliar frequencies were also unrewarded.

### Data analysis

For training sessions, total trial time, loudspeaker touch time (LST), and whether the choice was correct or incorrect was noted after each trial. Total trial time here is defined as the time from when the trial started by opening the SC until 10 s after leaving the SC. LST is defined as the time from when the shark’s head passed the opening of the starting compartment until the time the shark touched the black circle on the loudspeaker with its nose. As the trial time was capped at 10 s, if the black circle was not touched within this period, LST was noted as 15 s (Group 1) or 12 s (Group 2) (in order to clearly distinguish a touch at 10 s from not touching). To prove statistical significance of learning success, the probability of achieving the learning criteria by chance was determined as less than 5% (χ^2^ test, p < 0.05).

For transfer tests, total trial time and action (i.e., touching or not touching the loudspeaker) were recorded for each trial. Transfer data from Group 1 was visualized, but due to the small amount of data no statistical tests were run. For Group 2, Transfer 1 data was analysed by using Generalized Linear Mixed Models (GLMM) to model action per trial type. Transfer test type was included as a fixed effect and individual as a random effect to account for individual variation. A GLMM was also used to determine whether transfer test LST times differed from regular LST times with type of session as fixed effect and individual as random effect. Transfer 2 data was visualized, but no statistical tests were run.

Best fitted models were selected based on the dispersion plots and Akaike’s Information Criterion (AIC) value (SI 1). For all models, Wald Chi-square tests were performed to determine statistical significance of each effect. For all tests p < 0.05 was considered significant and p < 0.001 highly significant. All analyses and plots were run using the programming language R (v.4.0.3;^63^) in the software R Studio (v. 1.3.1073;^64^). Plots were produced using ‘ggplot2’^65^. Packages ‘lme4’^66^, ‘multcomp’^67^, ‘DHARMa’^68^ and ‘car’^69^ were used for statistical analyses.

### Ethics statement

The research reported here was performed under the guidelines established by the current German animal protection law. The experimental protocol for behavioural trials on fish was approved by the ethics committee of the LANUV (State Office for Nature, Environment and Consumer Protection North Rhine-Westphalia, Germany; AZ 81-02.04.2020.A432). All applicable international, national, and/or institutional guidelines for the care and use of animals were followed. The authors complied with the ARRIVE guidelines.

## Results

### Acoustic measurements

Sound pressure levels (SPL) for ambient sound, a 90 Hz and a 210 Hz tone were measured in the experimental tank along a 15 cm grid (Fig. 2). SPL varied throughout the tank, with ambient sound being lowest on average (range 97–101 dB re 1 µPa), 90 Hz being loudest in the upper left corner (range 97–128 dB re 1 µPa) and 210 Hz being loudest around the loudspeaker (range 93–135 dB re 1 µPa). SPL between the loudspeaker and SC (which is where the sharks swam most of the time) are comparable. SPL for all intermediate frequencies were only measured at grid point ‘i7’ (Table 2).

**Table 2:** SPL (in dB re 1 µPa) of Transfer 2 tones at grid point 'i7' in the experimental tank.

Tone (Hz)	100	110	120	130	140	150	160	170	180	190	200
Mean SPL	106.7	112.0	115.0	117.0	111.0	104.0	109.0	100.0	109.2	111.3	107.2
SE SPL	0.17	0.03	0.03	0.03	0.03	0.09	0.03	0.00	0.00	0.00	0.09

### Group 1

Group 1 consisted of six females and one male, ranging in size from 63 to 75 cm and were referred to as shark A–G. All animals were successful in learning the first two steps (step 1: touching the loudspeaker when 200 Hz is played; step 2: not touching the loudspeaker when no tone is played and touching when 200 Hz is played). In the actual discrimination task, where 140 Hz was used to indicate a ‘No-Go’ trial and 200 Hz to indicate a ‘Go’ trial, all four participating individuals were successful. Due to sharks being trained in a supersession method, no learning curve is provided.

#### Transfer tests

**Figure 5:**
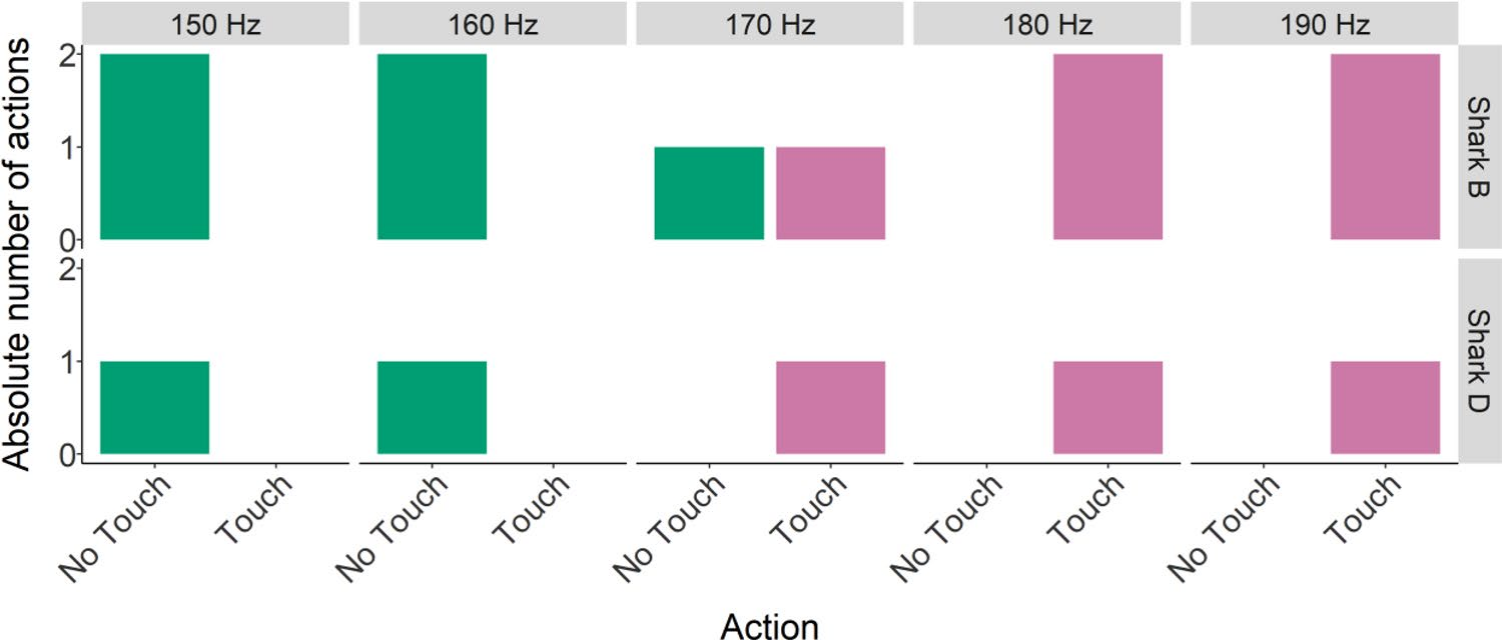
Grouped supersession data absolute number of action (not touching or touching the loudspeaker) per unfamiliar frequency (150–190 Hz).

After successfully learning the ‘Go/No-Go’, two animals participated in supersessions where unfamiliar frequencies were introduced to determine the difference threshold. Due to time constraints, shark B performed two transfer supersessions, and shark D only one. Both shark B and D started touching the loudspeaker (action A) at 170 Hz (Figs. 3, 4, 5), meaning the difference threshold for these sharks was 30 Hz.

### Group 2

**Figure 6:**
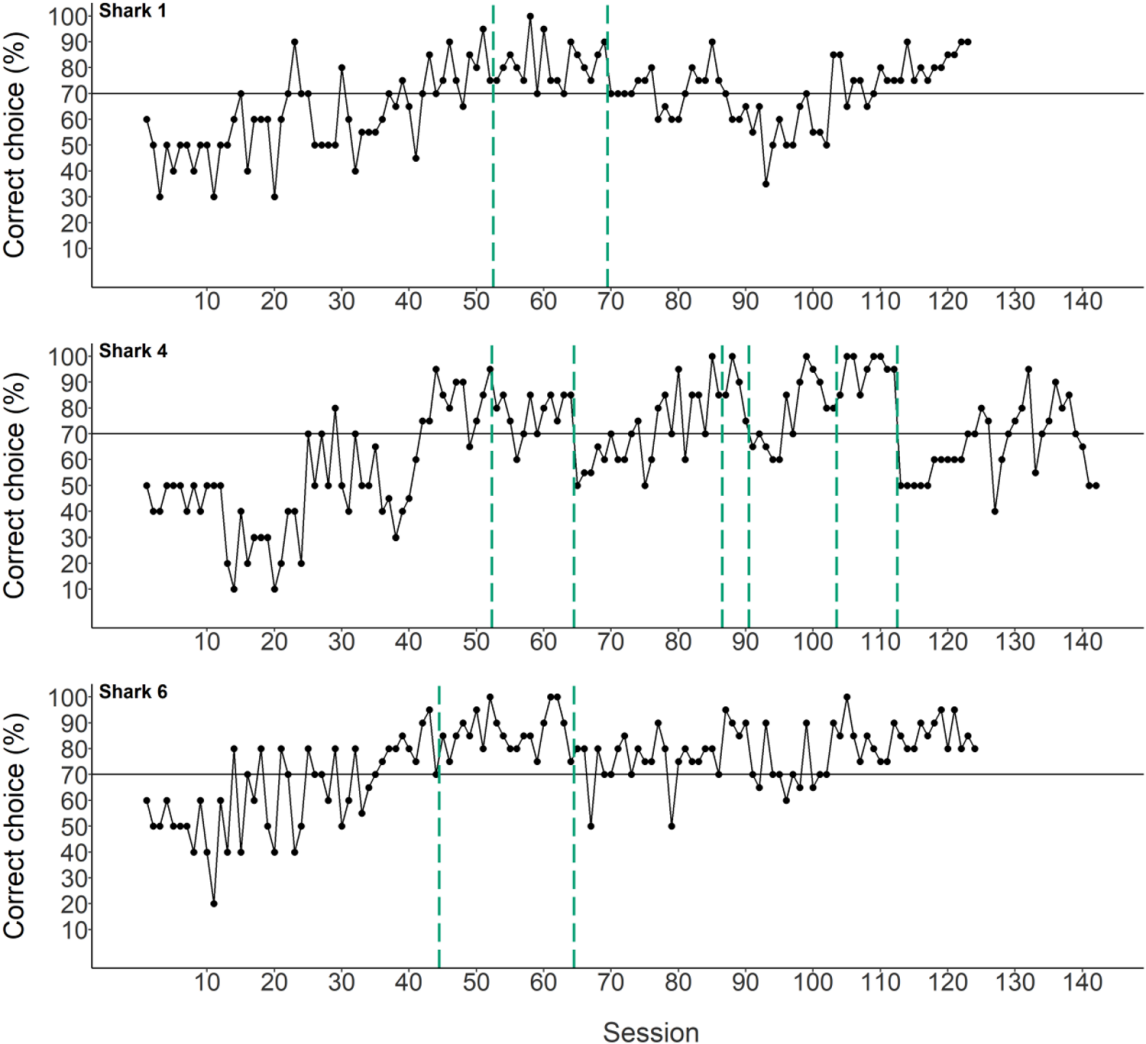
Individual data on percentage of correct choices (learning curve). The solid horizontal line indicates the 70% LC threshold. Vertical dashed green lines indicate sessions including transfer test trials for utilized stimuli. After completing the first set of transfer tests, the visual stimulus was taken out.

Due to time constraints, super sessions for group 1 were only performed three times. Since results were promising, a second experimental group was formed. Nine sharks started the experimental training procedure in Group 2. Acclimatization of the sharks was completed after four days, since eight out of the nine sharks swam uninhibited, ate from the forceps and were accustomed to the starting compartment and loudspeaker. Pre-training stopped when sharks swam out of the starting compartment voluntarily at least five times in a session. The following section will only summarize the results for those sharks that were successful in learning the task. Individual data of three sharks is presented, since participation of six sharks was stopped (one shark did not pass pre-training and did not progress to the training phase, and experimental participation of five sharks was stopped due to their lack of improvement or active participation in the experiment). The learning criterion in Part A (combination of visual and acoustic stimuli present) was reached by three sharks after 39 (shark 6) and 46 (sharks 1 and 4) sessions, respectively (Table 3). The learning criterion in Part B (only acoustic stimulus present) was reached by the same three sharks after the fifth (shark 1), eighth (shark 6) and 26th (shark 4) session (Table 3). Average LST time (per shark) during all sessions ranged from 5.9 to 6.3 s (average of all sharks: 6.0 ± 0.5 s). Learning curves, LST time and statistical analyses are discussed per individual (Fig. 6).

**Table 3:** Result of acoustic discrimination tests for part A (acoustic and visual stimulus present) and part B (acoustic stimulus present) in juvenile bamboo sharks. Number of sessions needed to reach the learning criterion (LC: 70% correct choices in five consecutive sessions) and average loudspeaker touch (LST) times [s]. Note that the average times are the same for part A and B.

Stimulus presented	Subject	Sessions to reach LC
A. Acoustic + visual stimuli	Shark 1	46
	Shark 4	46
	Shark 6	39
B. Acoustic stimulus	Shark 1	5
	Shark 4	26
	Shark 6	8

#### Transfer tests

##### Transfer 1. Utilized stimuli

**Figure 7:**
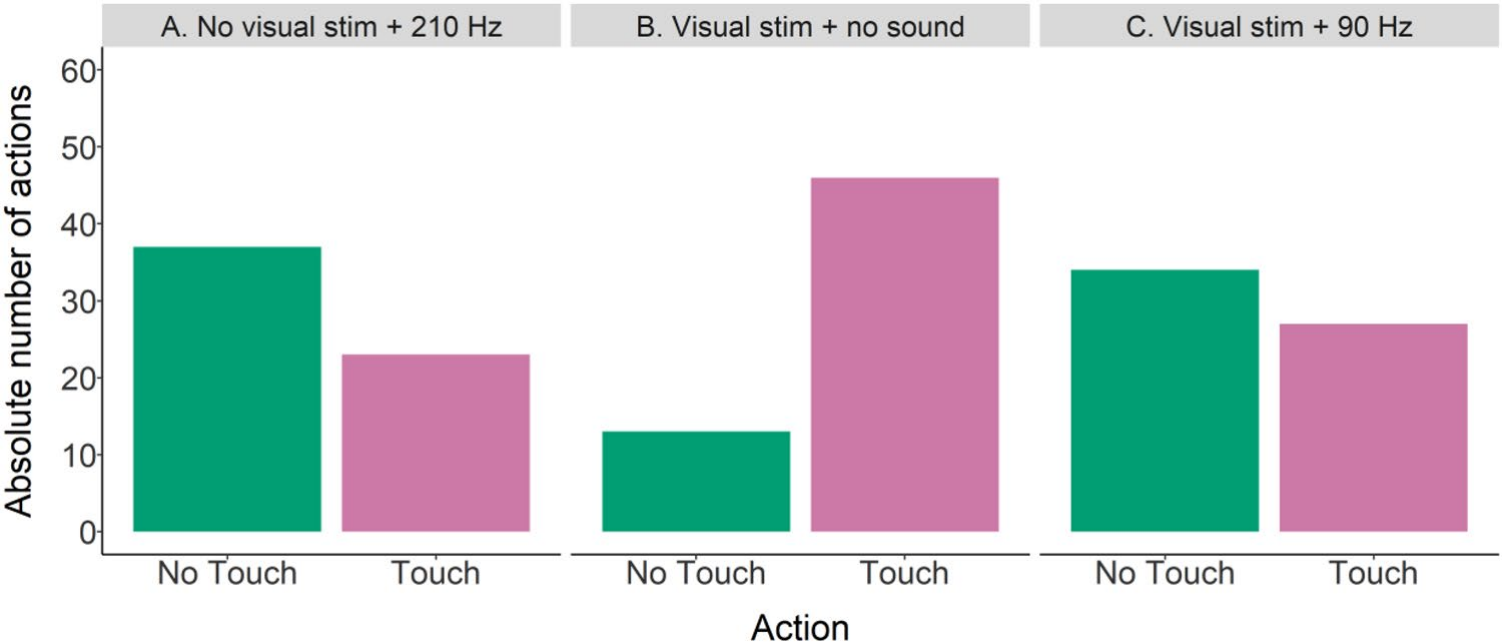
Grouped data (N = 3) for absolute number of action (not touching or touching the loudspeaker) and transfer type (A, B or C).

**Figure 8:**
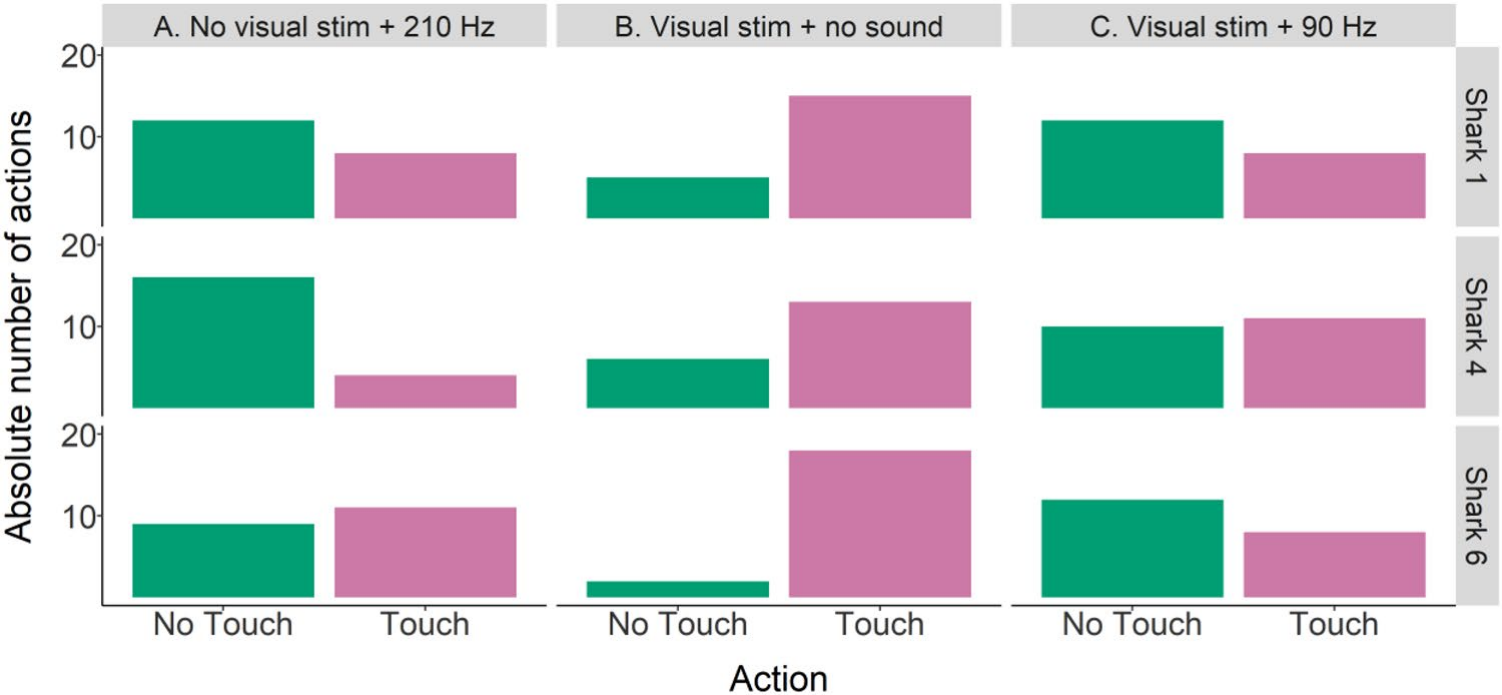
Individual data for absolute number of action (not touching or touching the loudspeaker) per transfer type (A, B or C).

No significant effect of individual on action (i.e., touching or not touching the loudspeaker) was found. However, due to the small number of individuals (N = 3) and observed variation between individuals, both grouped and individual data are shown (Figs. 7 and 8). There was a significant effect of type of transfer test on which action was performed (GLM_binomial_; χ^2^ = 20.0, df = 2, p < 0.001). In transfer type B (visual stimulus + no sound; Fig. 7) the loudspeaker was touched significantly more often than not (Tukey: z = 4.2, p < 0.001), indicating that sharks can use the visual stimulus alone as a cue for trial type. During type A trials (no visual stimulus, 210 Hz; Fig. 7) and type C (visual stimulus, 90 Hz; Fig. 7) one action was not performed significantly more often than the other, indicating that the conflicting information caused the sharks to not be able to choose and therefore act according to chance. However, looking at the performance of shark 4 in transfer type A (Fig. 8) there seems to be a notable difference in action: out of 20 transfer trials, he chose to not touch the loudspeaker 16 times versus touching the loudspeaker four times. No significant effect of type of session (regular or transfer test session) on LST time was seen.

##### Transfer 2. Difference threshold

**Figure 9:**
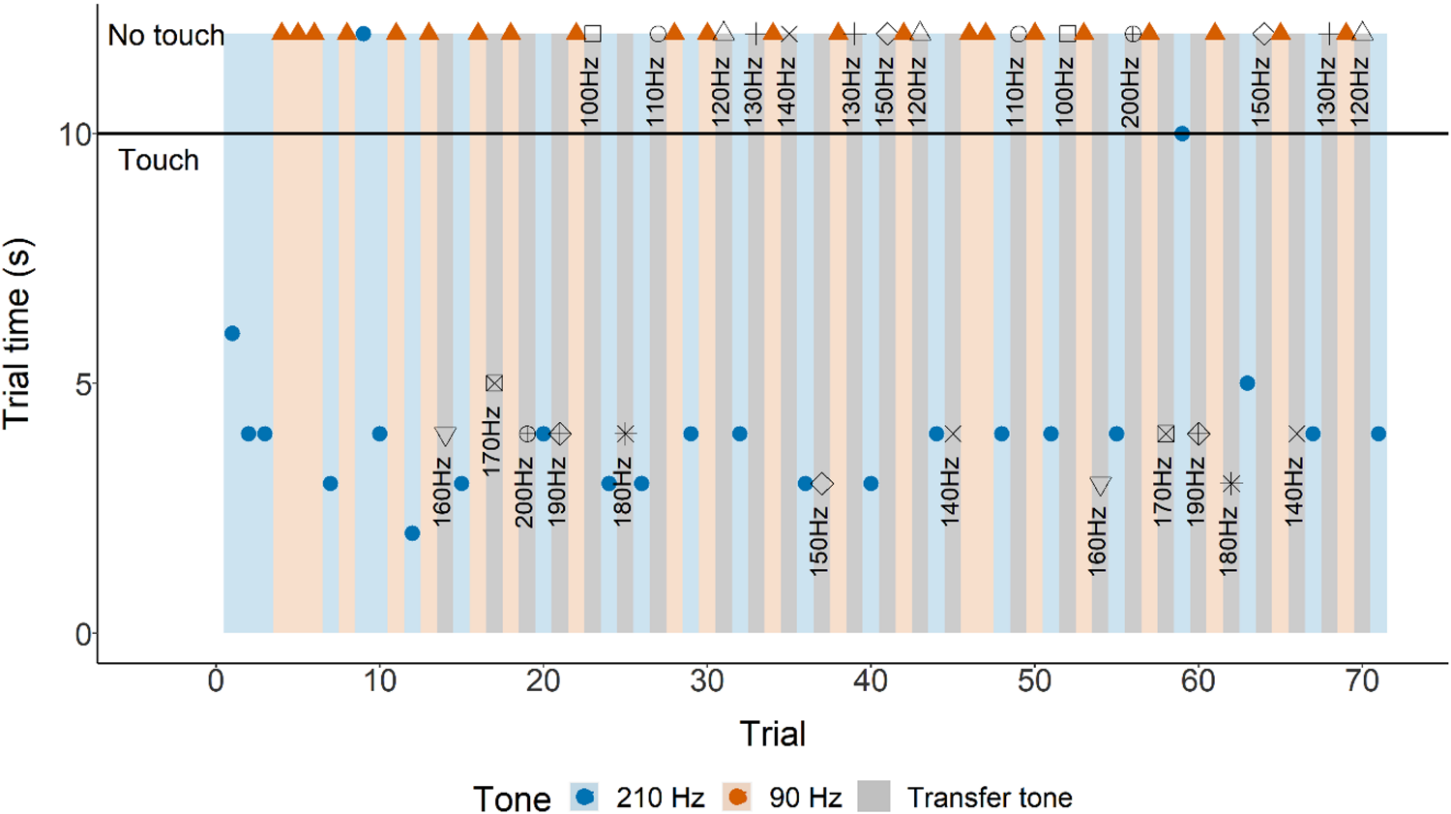
Supersession 10 (shark 1). Touching the loudspeaker indicated by the time it took to touch (LST time). Not touching the loudspeaker indicated by the shapes (at 12 s, indicating a capped trial i.e., the loudspeaker was not touched) above the horizontal solid black line (indicating the maximum trial time). Background colour indicates each separate phase (‘Go’ phase: blue, ‘No-Go’ phase: ‘orange, transfer: grey).

**Figure 10:**
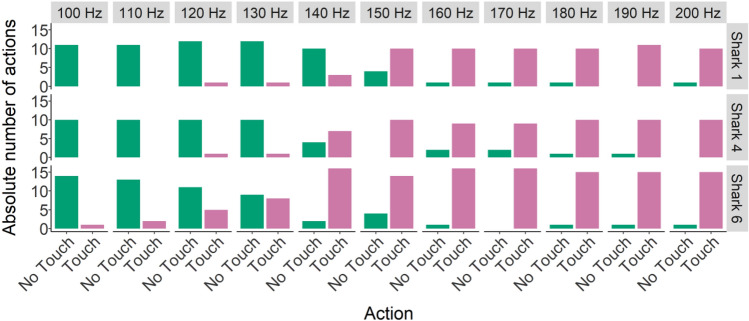
Grouped supersession data absolute number of action (not touching or touching the loudspeaker) per unfamiliar frequency (100–200 Hz).

After reaching LC in part B (only acoustic stimulus present), supersessions were conducted with all three individuals. Transfer tones were interspersed without a specific order. An example of the structure of these supersessions is presented in Fig. 9. Grouped data is shown in Fig. 10.

## Discussion

This study explored cognitive abilities of the grey bamboo shark *C. griseum* by conducting acoustic discrimination experiments on 16 juvenile sharks with a Go/No-Go method, never before used on sharks. The results showed that grey bamboo sharks can perform a Go/No-Go task and seven sharks were able to perform this task based on acoustic stimuli. Transfer tests further elucidated that when both visual and acoustic stimuli were presented together, both types of cues were used. This indicates that both visual and acoustic cues were acquired during training. The smallest difference between frequencies that sharks successfully discriminated between was 30 Hz.

### Acoustic discrimination

Seven sharks successfully learnt the Go/No-Go task, showing that bamboo sharks can perform multiple types of cognitive tasks such as two-alternative forced choice^1^ and Go/No-Go tasks. In Group 1, four sharks reached LC. In Group 2, sharks were fully accustomed to the experimental set-up by the time the experiment commenced, and three sharks reached learning criterion after 39 and 46 sessions. Reaching LC required more sessions than learning other visual tasks in previous experiments with this species (between 10–30 sessions)^7,13,70^.

Grey bamboo sharks have been highly successful in learning visual discrimination tasks in previous studies. Vision seems to be a primary sensory system for fish, including sharks, living in tropical clear water environments^71,72^. Based on this and other studies^73^ sharks were expected to prefer a visual stimulus over an acoustic one when both were presented simultaneously. However, this was not the case. While the visual stimulus may have been essential for learning to occur, it was not preferred as the deciding cue in Transfer A and C, i.e., sharks did not touch the loudspeaker significantly more often than not. This shows that both the acoustic and visual stimulus had been learnt during training and that the conflicting information in the transfer test situation caused the sharks to choose according to chance.

For Transfer 1 from Group 2, both grouped and individual data was shown even though according to statistical analysis there was no effect of individuals on the model. When looking at the actual data, a notable difference in action in type A was seen in shark 4. After Transfer 1, the use of the visual stimulus was stopped, and only acoustic stimuli were presented. Two of the sharks (shark 1 and 6) were quick to catch on and reached learning criterion within the first week, indicating that the acoustic cues had already been associated with the respective actions. In contrast, shark 4 took over four weeks (26 sessions) to reach learning criterion again. The difference in action in transfer type A for shark 4 and the long time it took shark 4 to re-learn the acoustic task indicate that it was more focused on the visual stimulus (and therefore confused by the lack of it after having been removed) than the others. Another option is, that differences in the sharks' personalities accounted for the different behaviors^74^, highlighting the importance of taking individual variation into account.

### Difference threshold

In the lower frequency range (90–110 Hz) that was tested in Group 2, it seems that a difference of 30 Hz between the target frequency  (90 Hz) and an unfamiliar frequency was sufficient to differentiate between them. This means that a clear difference in behavioural response was seen when playing 90 Hz or, for example, 120 Hz. In contrast, it seems that a similar difference of 30 Hz in the higher frequency range (160–210 Hz) did not elicit a change in action performed. However, results from Group 1 showed that 200 Hz can still be successfully differentiated from 170 Hz. Thus, a difference of 30 Hz was also recognized in the higher frequency range (160–210 Hz). In a previous study, sharks were taught to perform a two-alternative forced choice experiment using 30 vs. 80 Hz and later 140 vs. 200 Hz^73^. Transfer testing showed there was a greater difficulty in assigning new frequencies in the lower frequencies (around 30 Hz) and higher frequencies ranges (around 200 Hz) than the tested mid-range frequencies (80–140 Hz)^73^. These findings support the results of Group 2 from this study. Our results indicate that sharks’ ability to discriminate new frequencies in the range of 90–110 Hz is higher than in the range of 160–210 Hz. Training session methods differed within this study, but the experimental procedure for determining the difference threshold was similar and we assume results are therefore comparable. These result are also in line with previous research on Port Jackson sharks *H. portusjacksoni*, which showed that sharks could learn an association task using an acoustic stimulus^21^, and lemon sharks *N. brevirostris*, which showed that sharks could correctly perform a discrimination task using 60 vs. 40 Hz (i.e. a difference of 20 Hz)^20^. Results of studies specifically looking at just noticeable differences (JND; a measure frequently used for studying discrimination thresholds) in teleosts found comparable results. JND for goldfish *C. auratus* was 9.4 Hz and for electric fish (Pollimyrus) 8.7 Hz^37,40^. Further testing is needed to determine whether the action performed in the higher frequency range is consistent when repeated multiple times, and what animals choose when frequencies outside of the 90–210 Hz range are presented.

### Implications of the study

Anthropogenic noise is considered a global source of environmental pollution^75^ and ranges anywhere from 0 to over 10,000 Hz, with median noise levels occurring between ca. 50–500 Hz^76–78^. With the exception of high-frequency sonar, most anthropogenic noise thus overlaps considerably with the hearing ranges of sharks and other marine organisms and can cause severe problems both behaviourally as well as physiologically. Although potential effects are presently unknown, new or unnatural sounds are likely to interfere with shark behaviour. Recent studies have shown that in areas where either an artificial sound or playback of an orca-call was played, fewer reef and coastal sharks appeared compared to silent control trials^49^. Sound was also shown to deter Port Jackson sharks *H. portusjacksoni* and epaulette sharks *Hemiscyllium ocellatum* from responding to a positive stimulus such as food^52^. These findings agree with findings in southern stingrays *Hypanus americanus*, who altered their swimming behaviour in response to tones ranging from 50–500 Hz^79^.

The specific impact of noise depends on the nature of the sound and whether the receiving organism is responsive to either particle motion, sound pressure or both^80^, with most bony fishes being able to detect both. Research studying the effects of anthropogenic noise on bony fishes has shown that it can impact fishes both on an individual as well as a population level. Anthropogenic noise is harmful to marine organisms by impacting natural behaviour and can cause physical damage^23^ and potentially even death, either directly or indirectly^81,82^. Anthropogenic noise affects individual fish by causing hearing loss^83,84^, inducing stress^83,85–87^, impacting immunity^88^ and/or changing reproductive, social or other behaviour such as orientation^83,89–95^. Moreover, a recent study on reef fish showed that sound pollution interferes with learning to recognize a novel predator^96^. This is especially concerning considering that the capacity of learning allows an organisms to adjust their behaviour based on previous experiences and environmental variation^1,4^. On a population level, anthropogenic noise can disrupt the collective dynamics of fish shoaling^97^ and affects population growth by impairing survival and reproduction^82,83,86,89,94,98^. With anthropogenic noise levels predicted to increase, a better understanding of the use of sound by sharks and the potentially interfering effects of sound pollution on sharks and their learning abilities is imperative for proper management and protection.

## Conclusion

The data presented in this study reveals the ability of grey bamboo sharks to perform a Go/No-Go task using both visual and acoustic stimuli. No clear preference for the type of the presented sensory cues was detected, although individual variation was seen. The frequency difference threshold of 30 Hz found in this study is comparable to results of previous experiments. Due to the small sample size, results of the statistical analysis should be considered with caution and individual results should be concentrated on. Many cognitive studies on sharks so far have used visual stimuli. Considering the poor knowledge of the use of sound by sharks and anthropogenic noise expected to rise, new insights into sound recognition, detection and use are crucial to the management of healthy populations of sharks.

## Supplementary Information

Supplementary Information.
