# Supplementary material for: Acoustic discrimination in the grey bamboo shark Chiloscyllium griseum
Source: Sci Rep. 2022 Apr 20;12:6520. doi: 10.1038/s41598-022-10257-1 (PMC9021286; doi:10.1038/s41598-022-10257-1)
Supplement: Supplementary file 1 — Supplementary Information. [file 41598_2022_10257_MOESM1_ESM.docx]

Acoustic discrimination in the grey bamboo shark *Chiloscyllium griseum*

Tamar Poppelier^1^, Jana Bonsberger^1^, Boris Woody Berkhout^2^, Renée Pollmans^1^ & Vera Schluessel*^1^

^1^Department of Comparative Sensory Biology and Neurobiology, Institute of Zoology, University of Bonn, Meckenheimer Allee 169, 53115 Bonn, Germany

^2^ FNWI, University of Amsterdam, Science Park 904, 1098 XH Amsterdam, Netherlands

*Corresponding author: Vera Schluessel v.schluessel@uni-bonn.de

## **Supplementary information**

### **SI 1. Best fitted models**

Table 1. Best fitted models and AIC values of Group 2 Transfer 1 models.

|  | **Model** | **AIC value** |
| --- | --- | --- |
| *Grouped data* | Action ~ ID*Type | 234.6 |
| *Individual data* | Action ~ Type + (1\|ID) | 233.9 |

### **SI 2. Spectrograms**


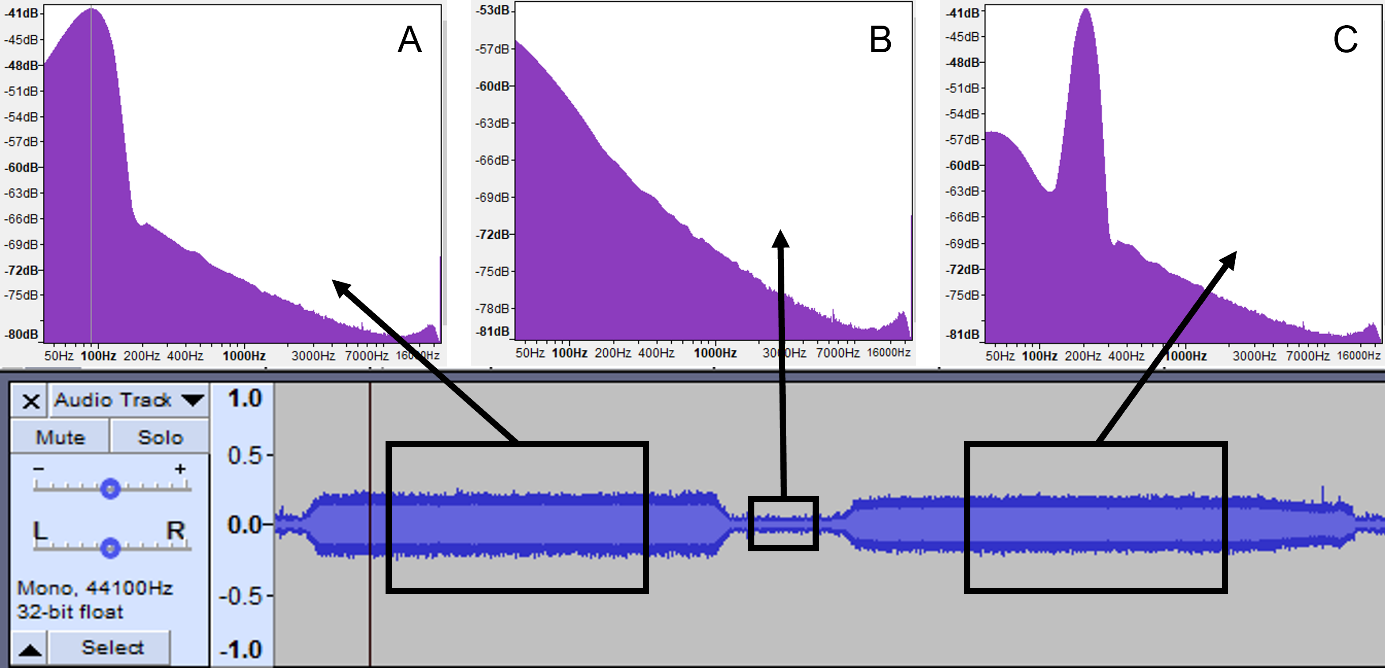


Figure 1. Examples of spectrograms and recorded traces of 90 Hz (A), ambient sound (B), and 210 Hz (C) at grid point ‘i7’.
